# Sleep Misperception and Associated Factors in Patients With Anxiety-Related Disorders and Complaint of Insomnia: A Retrospective Study

**DOI:** 10.3389/fneur.2022.836949

**Published:** 2022-04-07

**Authors:** Yingjie Liang, Xu Zhao, Changyong Zhang, Guangya Liu, Baili Lu, Li Han, Fang Tong, Xinyu Luo, Chuang Hu, Hui Liu

**Affiliations:** ^1^Department of Psychiatry, Wuhan Mental Health Center, Wuhan, China; ^2^Department of Sleep Disorder, Wuhan Hospital for Psychotherapy, Wuhan, China; ^3^Department of Psychiatry, Wuhan Wudong Hospital (Wuhan Second Mental Hospital), Wuhan, China; ^4^Outpatient Office, Wuhan Jinyintan Hospital, Wuhan, China

**Keywords:** misperception index, sleep misperception, anxiety-related disorders, polysomnography, total sleep time

## Abstract

**Purpose:**

Data on sleep parameters by polysomnography (PSG) in patients with anxiety-related disorders are limited. Although the disturbance and risk factors of sleep misperception have been implicated in psychopathology, its role in anxiety-related disorders remains unclear. This retrospective study aimed to explore the characteristics and sleep parameters in patients with anxiety-related disorders and different sleep perception types, and to explore the associated factors for sleep misperception.

**Methods:**

Patients with anxiety-related disorders who had complaint of insomnia for more than 3 months were collected at Wuhan Mental Health Center between December 2019 and July 2021. Patients underwent a two-night PSG monitoring and completed a self-reported sleep questionnaire. Behaviors were assessed using 30-item Nurses' Observation Scale for Inpatient Evaluation (NOSIE-30). Patients were divided into normal sleep perception (NSP), positive sleep perception abnormality [PSPA; overestimation of total sleep time (TST) >60 min], and negative sleep perception abnormality (NSPA; underestimation of TST >60 min) groups. PSG indicators and NOSIE-30 scores were compared among groups using the one-way analysis of variance and the Kruskal-Wallis test. Multiple linear regression analysis was performed to determine the associated factors for misperception index.

**Results:**

The subjective and objective TST were 5.5 ± 1.9 h and 6.4 ± 1.7 h in 305 patients, respectively. Sixty-nine (22.6%) had PSPA, 80 (26.2%) had NSP, and 156 (51.1%) had NSPA. Subjective TST and objective sleep parameters were significantly different among groups. No statistical differences in NOSIE-30 subscale and total scores were observed among groups. Sex, rapid eye movement (REM)/TST (%), sleep efficiency, number of awakenings, Non-rapid eye movement of stage 2 sleep (NREM)/TST (%), REM spontaneous arousal times, sleep latency, diagnosis, social competence, and manifest psychosis were associated with sleep misperception.

**Conclusion:**

Sleep misperception is common in patients with anxiety-related disorders. Various sleep perception types have different PSG profiles, but similar personal and social behaviors. These data may be helpful to conduct personalized treatment.

## Introduction

Sleep is an important psychophysiological process for mental health (1). Sleep disturbance in mental disorders can negatively affect cognitive, emotional, and social functions (2). Anxiety-related disorders, as a branch of mental disorders, are characterized by abnormal maladaptive forms of emotional response to potential threat. The common types of anxiety-related disorders according to the fifth edition of Diagnostic and Statistical Manual of Mental Disorders (DSM-V) include generalized anxiety disorder, obsessive-compulsive disorder, social anxiety disorder, separation anxiety disorder, panic disorder, agoraphobia, and specific phobia (3). Increasing evidence demonstrates sleep disturbance in patients with anxiety-related disorders (4, 5), with a potentially bidirectional relationship. However, current findings in sleep profiles are mostly focused on individual anxiety-related disorders, and sleep assessment modality was inconsistent among studies (5). A comprehensive evaluation of sleep disturbance in this whole branch of mental disorders is needed.

Objective measures have been increasingly used for the analysis of sleep problems. However, an interesting phenomenon, discrepancy between subjective and objective sleep parameters, often appears. This sleep misperception is directly reflected in the divergence of subjective and objective total sleep time (TST), including underestimation and overestimation of TST. Patients will frequently go to the hospital due to dissatisfaction with sleep quality, seeking for solution. Sleep misperception has been implicated in many kinds of insomnia. The prevalence of sleep misperception in the sleep disorder population varied widely, ranging from 9.2 to 55.5% (6 7 9), due to different definitions of sleep misperception used. Polysomnography (PSG) is arguably the objective standard to assess sleep parameters. Although the diagnosis of insomnia is usually not based on objective parameters, it is necessary to use both objective and subjective methods for capturing the sleep experience and its characteristics. However, most of the current studies on sleep misperception excluded patients with mental disorders. Currently, only a small number of studies analyzed sleep misperception, with scarce data on subjective and objective sleep parameters in psychiatric patients. A study by Wenigmann et al. assessed psychiatric patients with sleep disorders, and sleep misperception was observed in 52% of the 249 examined patients (6). Bian et al. reported a sleep misperception rate of 43.2% in 148 patients with schizophrenia (7). Interestingly, 38.5% of patients with schizophrenia showed overestimation of their TST (7). However, sleep misperception with quantitative analysis of sleep parameters is limited in patients with anxiety-related disorders. Although a previous study preliminarily provided some evidence on sleep disturbance in anxiety-related disorders by collectively using meta-analysis and combining subjective and objective measures together, differences between subjective and objective data among patients with different sleep perception were not characterized. In addition, further studies are needed to identify the associated factors for sleep misperception.

This study aimed to explore the characteristics and sleep parameters in patients with anxiety-related disorders and different sleep perception types, and to explore the associated factors for sleep misperception. We hope to provide some reliable evidence for sleep assessment, clinical classification, and appropriate treatment of patients with anxiety-related disorders.

## Methods

### Patients

This was a retrospective study conducted in the Wuhan Mental Health Center between December 2019 and July 2021. The inclusion criteria were as follows: (1) age ≥18 years; (2) diagnosis of anxiety-related disorders according to DSM-V criteria (3); (3) stable disorder; (4) complaint of insomnia for more than 3 months; and (5) ability to understand the study objective and willingness to complete the assessments. The exclusion criteria were as follows: (1) severe anxiety, as indicated by Self-rating Anxiety Scale score ≥70 (8); (2) other DSM-V axis I disorders; (3) prior electroconvulsive therapy within 1 month; (4) cognitive impairment, as indicated by Mini-Mental State Examination (MMSE) score ≤27 (9); (5) abuse of drugs or other substances; (6) other significant sleep disorders according to the third edition of International Classification of Sleep Disorder (ICSD-3) (10), including obstructive sleep apnea hypopnea syndrome (OSAHS), rapid eye movement (REM) or non-REM (NREM) parasomnia, circadian rhythm sleep-wake disorders, and movement disorders (such as restless leg syndrome and periodic leg movement disorder); (7) have used any medicine or health care products to regulate sleep in the past 5 days; and/or (8) incomplete data. The study was approved by the Ethics Committee of Wuhan Mental Health Center (No.KY201908.28). Written informed consent was obtained from each patient before examinations.

### Data Collection

Demographics, diagnosis of anxiety-related disorders, and medication history (in the last 6 months) were collected during the interview with a psychiatrist, two board-certified neurologists, and an expert in sleep medicine. After interview, patients underwent PSG monitoring and completed a self-reported sleep questionnaire. Blood pressure was measured before and after PSG monitoring.

### Polysomnography Monitoring

Patients received two-night standard PSG monitoring using a Compumedics-Greal PSG monitor (Australia Compumedics Limited). To exclude other sleep disorders and first night effect, data during the second night were collected for analysis. All patients entered the monitoring room at 21:30 and were asked to schedule their sleep and wake-up time according to daily routine. The monitoring room was quiet and comfortable, with proper room temperature, humidity, and light. Psychoactive substances, such as alcohol, tea, and coffee, were not allowed. The monitoring started from 22:00 to 7:00 the next morning. Complete records of electroencephalogram, electrooculogram, electromyogram, electrocardiogram, thermal airflow sensor, nasal pressure transducer, chest and abdomen respiratory movement, limb movement, body position, blood oxygen saturation, and snore sensor indicators for 6 h or longer were deemed as a successful monitoring. Patients with unsuccessful monitoring needed to be monitored again at next night. Items were recorded and analyzed according to American Academy of Sleep Medicine (AASM) criteria, version 2.6 (11), including TST, sleep latency, sleep efficiency (TST/time in bed ×100%), the percentage of NREM sleep (N1, N2, and N3 period) and REM sleep to TST, wake after sleep onset (WASO), number of awakenings, arousal index, and spontaneous arousal times. Arousal from sleep was deemed present when there were limb movements or respiratory events with oxygen desaturation for ≥10 s. Spontaneous arousal was defined as that which was not caused by respiratory events, oxygen desaturation, or limb movement. All Participants were asked to stop taking any sleep-affecting medication at least 5 days prior to testing as judged by our sleep specialist.

### Self-Reported Sleep Questionnaire

After successful PSG monitoring, patients were instructed to fill a questionnaire with four questions: (1) What time did you go to bed last night? (2) How long did it take you to fall asleep last night? (3) What time did you wake up this morning? (4) How long did you sleep last night?

### Sleep Perception

There are no recognized diagnostic criteria for sleep perception. According to previous studies (12–14), sleep perception abnormality is defined as a more than 60 min difference between self-reported subjective TST and PSG-measured objective TST. Positive sleep perception abnormality (PSPA) is defined as subjective overestimation of TST (subjective TST—objective TST >60 min). Negative sleep perception abnormality (NSPA) is defined as subjective underestimation of TST (objective TST—subjective TST >60 min). Normal sleep perception (NSP) is defined as ≤60 min discrepancy between subjective TST and objective TST. Sleep perception is calculated as subjective TST/objective TST (15). Lastly, sleep perception is calculated as (objective TST—subjective TST) (17).

### Nurses' Observation Scale for Inpatient Evaluation

The 30-item Nurses' Observation Scale for Inpatient Evaluation (NOSIE-30) is a highly sensitive scale to assess the behavioral changes in patients with psychiatric disorders (16–19). A Chinese version of NOSIE-30 was used in this study (20), which comprises 7 subscales: social competence, social interest, personal neatness, irritability, manifest psychosis, retardation, and depression. The first three subscales indicate positive behaviors, while the last four reflect negative behaviors. Each item is scored using a 5-Likert scale ranging from 0 (never) to 4 (always). Higher score indicates a higher frequency of each particular behavior.

### Statistical Analysis

Statistical analysis was performed using SPSS 22.0 (IBM Corp., Armonk, NY, USA). Continuous variables were tested for normal distribution using Shapiro-Wilk test. Continuous variables, in accordance with normal distribution, were expressed as mean ± standard deviation and were compared among groups using one-way analysis of variance with *post-hoc* Bonferroni correction. Continuous variables with skewed distribution were expressed as median [interquartile range IQR)], and were compared among groups using the Kruskal-Wallis test with *post-hoc* Bonferroni correction. Categorical variables were expressed as frequency (percentage) and were compared among groups using chi-square test or Fisher exact test, where appropriate. Multiple linear regression analysis was performed to determine the associated factors for misperception index, and stepwise method was used for the selection of independent variables. *p* < 0.05 was considered statistically significant.

## Results

### General Information and Sleep Parameters in the Total Patients With Anxiety-Related Disorders

**Figure 1:**
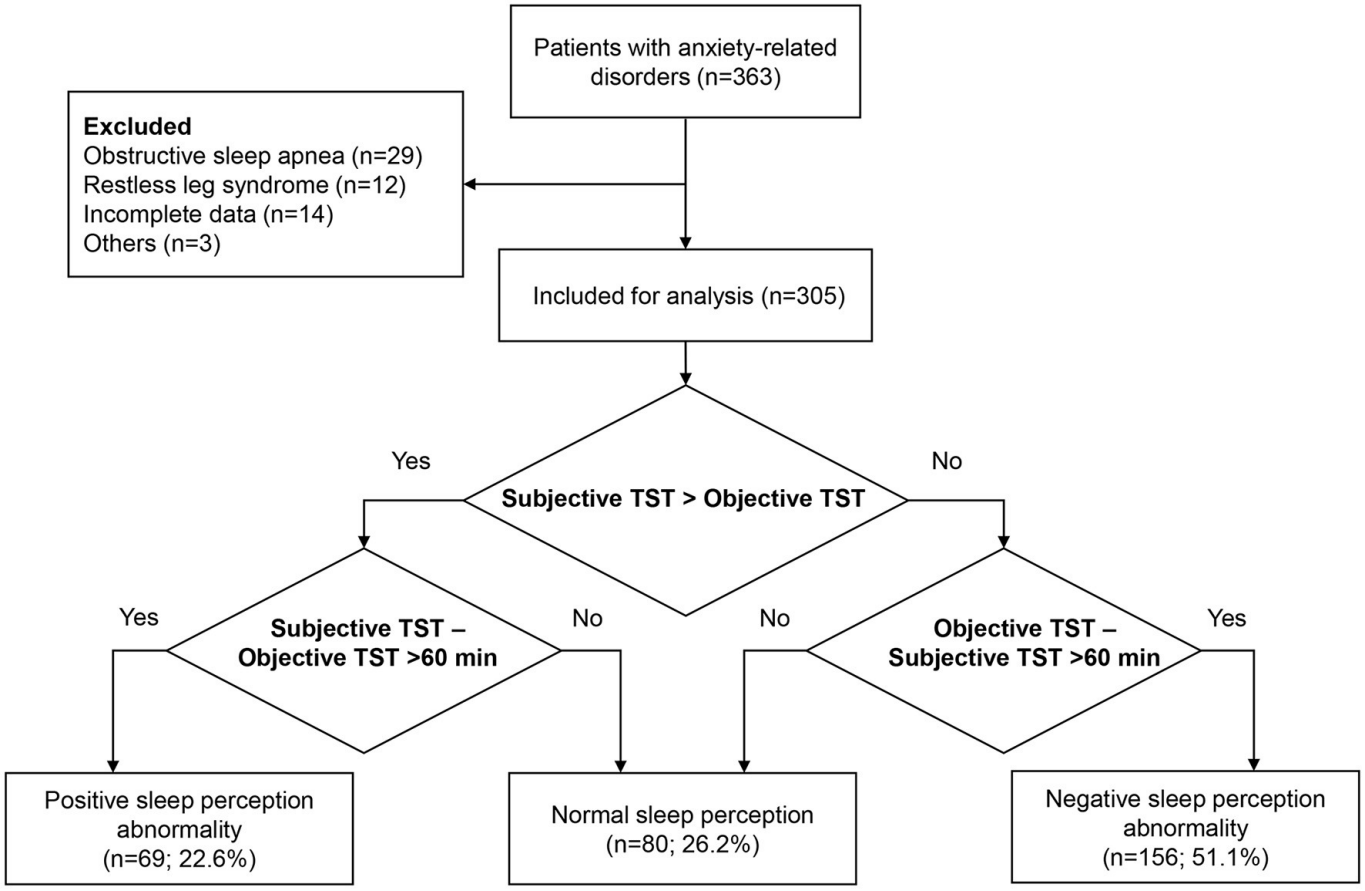
Study flowchart.

Between December 2019 and July 2021, a total of 305 patients who met the eligibility criteria with complete data were included in the analysis (Figure 1). The mean age was 44.0 ± 16.6 years, and 182 (59.7%) were females. Of 305 patients, 150 (49.2%) had generalized anxiety disorder, 81 (26.6%) had obsessive-compulsive disorder, 20 (6.6%) had mixed anxiety disorder, 15 (4.9%) had somatic symptom-related anxiety disorder, 14 (4.6%) had social anxiety disorder, 12 (3.9%) had separation anxiety disorder, five (1.6%) had hypochondriac anxiety disorder, four (1.3%) had panic disorder, and four (1.3%) had specific phobia. Sixty-nine (22.6%) had PSPA, 80 (26.2%) had NSP, and 156 (51.1%) had NSPA (Table 1). Regarding the sleep parameters in all the 305 patients, the subjective and objective TST were 5.5 ± 1.9 h and 6.4 ± 1.7 h, respectively. The sleep latency was 44.4 ± 38.5 min, and the sleep efficiency was 73.2 ± 18.6%. The misperception index was 0.1 ± 0.5 (Table 2).

**Table 1:** Patient characteristics.

**Characteristic**	**Total** **(*n =* 305)**	**PSPA** **(*n =* 69)**	**NSP** **(*n =* 80)**	**NSPA** **(*n =* 156)**	***P***
Age (years)	44.0 ± 16.6	46 (24, 57)	48 (34, 59)	46 (29, 56)	0.159
Sex					<0.01
Male	123 (40.3)	26 (37.7)	44 (55.0)	53 (34.0)	
Female	182 (59.7)	43 (62.3)	36 (45.0)	103 (66.0)	
BMI (kg/m^2^)	22.6 ± 3.6	23 (20, 24)	23 (21, 25)	22 (20, 25)	0.098
SBP before PSG (mmHg)	120.3 ± 14.9	119.5 ± 14.2	121.2 ± 13.1	120.2 ± 16.0	0.783
DBP before PSG (mmHg)	76.5 ± 11.2	75 (68, 81)	78 (71, 84)	75.5 (70, 82)	0.140
SBP after PSG (mmHg)	117.2 ± 14.5	114 (105, 121)	118.5 (112, 126)	117 (106, 125)	0.077
DBP after PSG (mmHg)	75.2 ± 9.6	72 (66, 80)	78 (72, 84)	75 (70, 79)	0.019
Medication history					
Sedative hypnotic drugs	198 (73.8)	36 (52.2)	57 (71.3)	105 (67.3)	0.035
Antidepressants	148 (48.5)	27 (39.1)	39 (48.8)	82 (52.6)	0.177
Mood stabilizers	42 (13.8)	10 (14.5)	8 (10.0)	24 (15.4)	0.514
Antipsychotic drugs	22 (7.2)	7 (10.1)	5 (6.3)	10 (6.4)	0.563
Antianxiety drugs	16 (5.2)	1 (1.4)	7 (8.8)	8 (5.1)	0.135
Diagnosis					<0.01
Generalized anxiety disorder	150 (49.2)	27 (39.1)	36 (45.0)	87 (55.8)	
Obsessive-compulsive disorder	81 (26.6)	9 (13.0)	23 (28.8)	49 (31.4)	
Mixed anxiety disorder	20 (6.6)	5 (7.2)	11 (13.8)	4 (2.6)	
Somatic symptom-related anxiety disorder	15 (4.9)	9 (13.0)	5 (6.3)	1 (0.6)	
Social anxiety disorder	14 (4.6)	11 (15.9)	2 (2.5)	1 (0.6)	
Separation anxiety disorder	12 (3.9)	5 (7.2)	0	7 (4.5)	
Hypochondriac anxiety disorder	5 (1.6)	1 (1.4)	1 (1.3)	3 (1.9)	
Panic disorder	4 (1.3)	2 (2.9)	0	2 (1.3)	
Specific phobia	4 (1.3)	0	2 (2.5)	2 (1.3)	

*Data are mean ± standard deviation, median (interquartile range), or n (%). BMI, body mass index; SBP, systolic blood pressure; PSG, polysomnography; DBP, diastolic blood pressure; PSPA, positive sleep perception abnormality; NSP, normal sleep perception; NSPA, negative sleep perception abnormality*.

**Table 2:** Polysomnography indicators, sleep perception, and misperception index.

**Item**	**Total** **(*n =* 305)**	**PSPA** **(*n =* 69)**	**NSP** **(*n =* 80)**	**NSPA** **(*n =* 156)**	***P***
Subjective TST (h)	5.5 ± 1.9	8 (7, 8)	6 (5, 6.3)^a^	4 (4, 6)^a^^b^	<0.01
Objective TST (h)	6.4 ± 1.7	4 (4, 6)	6 (5, 6)^a^	8 (7, 8)^a^^b^	<0.01
Number of awakenings (n)	64.1 ± 58.4	2 (1, 6)	57.5 (16, 96)^a^	76 (53, 111)^a^^b^	<0.01
Arousal index (per hour)	12.7 ± 14.5	4 (0, 10)	11.5 (6, 19)^a^	11 (8, 16)^a^	<0.01
Spontaneous arousal times (min)					
Total	40.8 ± 35.6	6 (3, 11)	37 (9, 60)^a^	52 (32, 72)^a^^b^	<0.01
NREM	38.7 ± 31.2	12 (9, 18)	32.5 (15, 54)^a^	47 (28, 64)^a^^b^	<0.01
REM	7.9 ± 9.4	11 (2, 21)	2 (1, 7)^a^	4 (1, 10)^a^	<0.01
Sleep latency (min)	44.4 ± 38.5	86 (78, 92)	50 (22, 81)^a^	13.5 (6, 32)^a^^b^	<0.01
N1/TST (%)	17.5 ± 15.3	10 (8, 18)	16 (13, 23)^a^	14 (9, 22)	<0.01
N2/TST (%)	77.0 ± 57.3	54 (28, 92)	70 (58, 91)^a^	64 (54, 73)	<0.01
N3/TST (%)	12.2 ± 10.9	14 (10, 19)	12 (3, 18)	8 (0, 17)^a^	<0.01
REM/TST (%)	29.3 ± 26.4	58 (47, 69)	14 (7, 49)^a^	15 (8, 20)^a^	<0.01
Sleep efficiency (%)	73.2 ± 18.6	60 (46, 76)	69.5 (58, 80)	85 (76, 91)^a^^b^	<0.01
WASO (min)	86.3 ± 68.7	72 (55, 129)	111.5 (66, 147)	54 (24, 79)^a^^b^	<0.01

*Data are mean ± standard deviation or median (interquartile range). Number of awakenings: the sum number of awake when falling asleep during the full night; Arousal index: the sum number of arousals ×60/TST (per hour); Spontaneous arousal times: the sum minutes of spontaneous arousal after falling sleep; TST, total sleep time; NREM, non-rapid eye movement; REM, rapid eye movement; WASO, wake after sleep onset; PSPA, positive sleep perception abnormality; NSP, normal sleep perception; NSPA, negative sleep perception abnormality.*

a *p < 0.0167 vs. PSPA by post-hoc Bonferroni correction.*

b *p < 0.0167 vs. NSP by post-hoc Bonferroni correction*.

### Comparisons of General Information, Sleep Parameters, and NOSIE-30 Scores Among Patients With Anxiety-Related Disorders and Different Sleep Perception Types

More females presented with PSPA or NSPA (*p* < 0.01) (Table 1). The lowest subjective TST and the highest objective TST were observed in the NSPA group [4 h (IQR, 4–6) and 8 h (IQR, 7–8)], followed by the NSP group [6 h (IQR, 5–6.3) and 6 h (IQR, 5–6)] and PSPA group [8 h (IQR, 7–8) and 4 h (IQR, 4–6)], with significant differences between each two groups after *post-hoc* Bonferroni correction (*p* < 0.01). Number of awakenings, total spontaneous arousal times, NREM spontaneous arousal times, sleep latency, sleep perception, and misperception index were also significantly different between each two groups (*p* < 0.01). Arousal index showed no statistical difference between the NSPA and NSP groups, but both were significantly higher than the PSPA group (*p* < 0.01). REM spontaneous arousal times and the percentage of REM sleep to TST showed no statistical differences between the NSPA and NSP groups, but all were significantly lower than the PSPA group (*p* < 0.01). The percentage of N1 period and N2 period to TST only showed significant differences between the NSP and PSPA groups (*p* < 0.01). The percentage of N3 period to TST only showed significant difference between the NSPA and PSPA groups (*p* < 0.01). Sleep efficiency and WASO showed no statistical differences between the NSP and PSPA groups, but all showed significant difference when compared with the NSPA group (*p* < 0.01) (Table 2). As shown in Table 3, there were no significant differences in subscale scores or total scores of NOSIE-30 among the three groups (*p* > 0.05).

**Table 3:** Results of the nurses' observation scale for in-patient evaluation (NOSIE-30).

**Item**	**Total** **(*n =* 305)**	**PSPA** **(*n =* 69)**	**NSP** **(*n =* 80)**	**NSPA** **(*n =* 156)**	***P***
Social competence	35.6 ± 4.1	36 (34, 38)	36 (34, 38)	36 (34, 38)	0.424
Social interest	16.5 ± 8.2	16 (12, 20)	16 (10, 20)	18 (10, 20)	0.996
Personal neatness	27.4 ± 3.8	28 (24, 28)	28 (24, 32)	28 (24, 32)	0.757
Irritability	8.5 ± 7.5	6 (0, 10)	8 (2, 12)	8 (4, 12)	0.135
Manifest psychosis	0.4 ± 1.6	0 (0, 0)	0 (0, 0)	0 (0, 0)	0.164
Retardation	3.9 ± 4.2	2 (0, 6)	4 (2, 6)	2 (0, 6)	0.111
Depression	2.9 ± 3.9	2 (0, 4)	2 (0, 6)	1 (0, 4)	0.687
Positive behaviors^a^	79.4 ± 11.8	80 (74, 86)	80 (70, 86)	80 (72, 86)	0.971
Negative behaviors^b^	15.7 ± 12.1	12 (6, 18)	16 (6, 24)	14 (6, 22.5)	0.136
Total score	191.9 ± 17.8	196 (186, 204)	192 (180, 204)	194 (182, 202)	0.532

*Data are mean ± standard deviation or median (interquartile range). PSPA, positive sleep perception abnormality; NSP, normal sleep perception; NSPA, negative sleep perception abnormality.*

a *Positive behaviors include the subscales of social competence, social interest, and personal neatness.*

b *Negative behaviors include the subscales of irritability, manifest psychosis, retardation, and depression*.

### Multiple Linear Regression Analysis of Associated Factors for Sleep Perception

The explanatory variable was defined as the “sleep perceptio*n* = objective TST–subjective TST.” Before performing multiple regressions, the first task was to conduct a variable screening. Using a forward stepwise regression method to select variables (age, sex, BMI, all variables shown in Tables 2, 3), the selection criteria was that significance-level *p*-value was <0.05. If the *p*-value is >0.05, the variable will be eliminated. The final retained variables were sex, REM/TST, sleep efficiency, number of awakenings, Non-rapid eye movement of stage 2 sleep (NREM)/TST (%), REM spontaneous arousal times, sleep latency, diagnosis, social competence, and manifest psychosis. If multiple collinearity tests (VIF, Variance Inflating Factor) are >5, it indicates a collinearity problem among variables. Otherwise, there is no collinearity problem among variables. Table 4 shows the regression results. Moreover, from the analysis of Table 4, we found that the VIF statistic of each explanatory variable was <5, indicating that there was no multiple collinearity problem among the variables, which was in line with the assumption of multiple linear regression. Then, Durbin-Watson (DW)test statistics was used to test whether the regression residue is self-phase when the DW statistic approached 2, indicating that the residue is not autocorrelated. The regression results showed that the DW was 1.920, close to 2, indicating that the residue has no autocorrelation.

**Table 4:** Multiple linear regression analysis of associated factors for sleep perception.

**Variable**	**Beta [95%CI]**	***p***	**VIF**
C	−0.392 [−2.582, 1.799]	0.725	–
REM/TST (%)	−0.039 [−0.048, −0.031]	<0.01	1.709
sex	0.465 [0.097, 0.833]	0.013	1.041
Sleep efficiency (%)	0.051 [0.04, 0.062]	<0.01	1.377
Number of awakenings (n)	0.007 [0.003, 0.011]	<0.01	1.519
N2/TST (%)	0.006 [0.003, 0.01]	0.001	1.257
Spontaneous arousal times of NREM (min)	−0.034 [−0.054, −0.013]	0.001	1.147
Sleep latency (min)	−0.01 [−0.017, −0.004]	0.002	1.913
Diagnosis	−0.063 [−0.122, −0.003]	0.039	1.098
Social Competence	−0.057 [−0.103, −0.01]	0.017	1.125
Manifest psychosis	−0.135 [−0.251, −0.018]	0.024	1.064
Adj-*R*^2^		0.663	
*F*		60.855 (*p* < 0.01)	
DW		1.920	

*TST, total sleep time; REM, rapid eye movement; WASO, wake after sleep onset; NREM, non-rapid eye movement*.

The adjusted goodness-of-fit *R*^2^ was 0.663, indicating that each explanatory variable can explain 66.3% of the information volume of the explained variable, and more than half of the information can be explained, indicating a good fitting effect. The model gameplay test *F* statistic was 60.855, and the significance-level *p*-value was <0.05, suggesting that the established multiple linear regression was valid.

Finally, multiple linear regression analysis showed that the sleep perception was associated with sex, REM/TST, sleep efficiency, number of awakenings, N2/TST, REM spontaneous arousal times, sleep latency, diagnosis, social competence, and manifest psychosis (Table 4).

## Discussion

This study compared clinical characteristics, sleep parameters, misperception index, and behaviors among patients with different sleep perception types (PSPA, NSP, and NSPA). Subjective TST and all the objective sleep parameters were significantly different among the three groups. Interestingly, although there were differences in the diagnoses of anxiety-related disorders among groups, similar personal and social behaviors were observed by NOSIE-30. Regarding results of the linear regression, younger age, more REM spontaneous arousal times, higher sleep latency, lower percentage of N3 period to TST, higher sleep efficiency, and higher WASO were associated with the increase in misperception index.

In our study, all the 305 patients were diagnosed with anxiety-related disorders, with the overall subjective TST of 5.5 ± 1.9 h and objective TST of 6.4 ± 1.7 h. This is consistent with previous studies that claimed that there is a universal difference between subjective and objective sleep time for all insomnia patients (21–23). Subjective sleep disturbance, decreased TST, and sleep continuity are robust in patients with anxiety-related disorders, with minor evidence for diminished sleep depth (5). However, studies that directly examined the distribution of different sleep perception types among patients with anxiety-related disorders are scarce. Underestimation of TST is prevalent among general insomniacs with normal objective TST (14, 21). In addition, some insomniacs can accurately estimate sleep time, while some may overestimate their TST (14). This phenomenon is also observed in our study of patients with anxiety-related disorders, of whom 51.1% underestimated their sleep time, 22.6% accurately estimate sleep time, and 26.2% overestimated their sleep time. The proportion of patients with normal sleep perception in our study was largely lower than general insomniacs and other psychiatric patients with sleep disorders (6, 7, 21). This indicates that patients with anxiety-related disorders who complain of insomnia may have worse sleep misperception. Pre-sleep cognitive activity is common in patients with anxiety-related disorders which are associated with underestimation of sleep time (5). These patients might also have paranoid ideation. Despite that 67.3% of patients in the NSPA group had used sedative hypnotic drugs, they still underestimated the sleep time. However, the high use rate of sedative hypnotic drugs seems not entirely useless. There were a certain number of patients with NSP or overestimation of TST in our study, which might be attributed to the effect of sedative hypnotic drugs. It was also possible that patients who overestimated their sleep time had potential cognitive impairment. A similar situation was observed in a study by Bian et al. (7), which showed that 43.2% of patients with stable schizophrenia presented sleep misperception, including 38.5% with overestimation of TST and 4.7% with underestimation of TST (7). Patients with schizophrenia tend to exaggerate their sleep quality, even for those with stable disease after effective antipsychotics treatment (24). Brain structure abnormality and alterations in neurotransmitters related to time perception may exist in patients with schizophrenia. In addition, COMT and ANKK1 genes are considered to be the possible reasons that lead to time misperception in these patients (25). Further research is needed to confirm whether neurophysiological mechanisms also exist in patients with anxiety-related disorders which contribute to sleep misperception.

In this study, we found that the sleep structure was completely different among groups. Patients with underestimation of TST showed lower sleep latency and WASO, but more NREM spontaneous arousal times and higher sleep efficiency than those with overestimation of TST or NSP. A study by Liu et al. grouped patients with OSAHA using the same method and showed similar results with our study in sleep structure among groups (12). Although patients in the NSPA group entered the sleep cycle faster, they had more spontaneous arousal times during the NREM period. They had difficulty in entering the REM period and would wake rapidly, with lower percentage of REM period to TST and WASO. Thus, they might believe that they get insufficient sleep, owing to decreased sleep depth and poor sleep continuity. Frequent periodic electroencephalogram changes during NREM period might be the reasons for sleep misperception (26, 27), but this needs further research. Despite the different sleep structure among groups, no differences were observed in personal and social behaviors. Some previous studies also analyzed the impact of sleep misperception on psychiatric symptoms, but the results are controversial (7, 14, 22, 28). The association of sleep misperception with psychiatric symptoms needs further investigation.

Identifying associated factors can help us carry out personalized treatment in risk population, which is also one of the objectives of our study. Sex, REM/TST (%), sleep efficiency, number of awakenings, N2/TST (%), REM spontaneous arousal times, sleep latency, diagnosis, social competence, and manifest psychosis were associated with sleep misperception. This was expected and consistent with the interplay of sleep structural parameters and was supported by previous studies (5, 12).

Although patients with insomnia often show a discrepancy between self-reported and objective sleep parameters, none of the standard treatment has been identified for insomnia have specifically targeted misperception of sleep. In our study, 73.8% of patients used sedative hypnotic drugs with high frequency prescription for insomnia in the last 6 months. Unnecessary use of sedative hypnotic drugs may bring side effects or lead to drug addiction syndrome and disrupt the integrity of sleep. Hence, treatment for self-reported sleep disorders should be reconsidered. Sleep misperception can be improved after psychotherapy (such as cognitive-behavioral therapy) for patients with insomnia (29). This might also be feasible in patients with anxiety-related disorders without any side effects, which deserves further investigations.

To our knowledge, this is the first study that explored the discrepancy between objective measures and subjective experience among patients with anxiety-related disorders in a large-scale study in China. However, there are still some limitations. First, potential bias was inevitable due to the retrospective nature and single-center design. Second, no data of imaging and pathological examinations were available in this study. Finally, only a two-night PSG was performed. Sleep time and misperception data obtained during multiple nights (such as seven nights) or by averaging might provide more valuable information with little variability.

In conclusion, this study indicates that sleep misperception is prevalent in patients with anxiety-related disorders. Objective sleep parameters differed in patients with various sleep perception types, with similar personal and social behaviors. Our results provide quantitative evidence for further study of sleep misperception in patients with anxiety-related disorders.

## Data Availability Statement

The original contributions presented in the study are included in the article/supplementary material, further inquiries can be directed to the corresponding author/s.

## Ethics Statement

The studies involving human participants were reviewed and approved by the Ethics Committee of Wuhan Mental Health Center. The patients/participants provided their written informed consent to participate in this study.

## Author Contributions

CZ and GL were involved in study conception and design. YL, XZ, BL, LH, FT, XL, CH, and HL were involved in the acquisition of data. YL and XZ were involved in the analysis and interpretation of data and were involved in drafting the manuscript. All authors were involved in revising the manuscript. All authors have also read and approved the final manuscript.

## Funding

This work was supported by the General Program of Wuhan Medical Research (Grant Number: WG20C03). The funding source had no role in the study design, collection, analysis, and interpretation of data, writing of the manuscript, or decision to submit the article for publication.

## Conflict of Interest

The authors declare that the research was conducted in the absence of any commercial or financial relationships that could be construed as a potential conflict of interest.

## Publisher's Note

All claims expressed in this article are solely those of the authors and do not necessarily represent those of their affiliated organizations, or those of the publisher, the editors and the reviewers. Any product that may be evaluated in this article, or claim that may be made by its manufacturer, is not guaranteed or endorsed by the publisher.
